# Contemporary management of treatment-related dermatologic toxicities in gynecologic cancers: a systematic review based on evidence from 2021 to 2025

**DOI:** 10.3389/fmed.2026.1751349

**Published:** 2026-02-20

**Authors:** Shanshan Wang, Rui Hou, Ruizhi Wang, Chu Liu, Shida Xu

**Affiliations:** Shenzhen Luohu People’s Hospital, Shenzhen, China

**Keywords:** 3D printed radiotherapy devices, chemotherapy, gynecologic cancer, paclitaxel, radiotherapy, skin management, skin toxicity, subclinical skin damage

## Abstract

**Introduction:**

Gynecologic cancers are commonly managed with chemotherapy, radiotherapy, and targeted therapies, which are effective but frequently associated with significant skin side effects. These dermatologic toxicities substantially reduce patients’ quality of life and treatment adherence, highlighting the need for a comprehensive synthesis of the latest evidence to address this clinical challenge.

**Methods:**

This systematic review summarizes the most up-to-date evidence (as of 2025) regarding treatment-induced skin damage in patients with gynecologic cancers. It focuses on synthesizing data related to the clinical manifestations, subclinical changes, risk factors, and management strategies of these skin toxicities.

**Results:**

Key findings include three critical areas of focus: subclinical skin damage (early, mostly asymptomatic changes induced by medications such as paclitaxel), radiotherapy-related skin issues, and severe skin effects associated with novel targeted therapies. Additionally, innovative technologies (e.g., 3D printed radiotherapy devices) were identified as potential tools for preventing and mitigating dermatologic toxicities.

**Discussion:**

The review emphasizes the importance of individualized intervention plans and multidisciplinary care models to effectively manage treatment-related skin toxicities. By synthesizing the latest evidence on manifestations, mechanisms, risk factors, and innovative mitigation strategies, this review aims to provide clinicians with optimal information to improve outcomes for gynecologic cancer patients experiencing skin toxicities, thereby enhancing treatment adherence and overall patient well-being.

## 1 Introduction

Gynecological cancers comprise cervical, ovarian, and vaginal cancers; these are considerable health issues for the female population across all parts of the world. These cancers significantly increase morbidity and mortality rates, and their management remains a major clinical challenge. Epidemiological studies from different areas, such as Japan and the US, show the presence and different clinical presentations of these cancer types. For example, extensive tumor registries run by the Japan Society of Obstetrics and Gynecology reported thousands of yearly cases, with most of the cases being cervical, endometrial, and ovarian cancers (1–6). Survival outcomes vary widely depending on cancer type and stage, with 5-year survival rates dropping considerably as the disease advances. Furthermore, there are racial and ethnic disparities that affect the results connected with gynecological cancers. It is clearest when we look at potential years of life lost (PYLL) and premature mortality rates in the US, which show that non-Hispanic Black women have far higher mortality rates and die much earlier than other populations (7, 8). These differences show that it is important to have special health programs for everyone and for people to be able to see doctors easily.

Given these epidemiological complexities, the management of gynecological malignancies involves an often integrated regimen of surgery, chemotherapy, radiotherapy, targeted therapy, and immunotherapy. In recent years, more treatment methods have become available. Molecular target therapy and ICI (immune checkpoint inhibitor) have emerged. This change in clinical management has improved the survival of patients and controlled the disease. However, these developments come with a set of treatment-related side effects, and skin side effects stand out as the most unacknowledged. Skin toxicities, such as rashes, pruritus, dry skin, and paronychia, and severe immune-related dermatological conditions, such as SJS and BP, can make a person’s life much worse, make it hard for them to keep taking their medicine, and even make it harder for them to fight off the cancer. Skin toxicities of gynecologic cancer patients are very important in the clinic, but it has been neglected for a long time, as it was considered that skin toxicities could be dealt with easily and the main target of treatment was to control the tumor.

Skin toxicities have gotten a whole lot more complicated with all these new treatments now in the mix. Targeted agents, such as EGFRIs and TKIs, typically cause cutaneous adverse effects by way of the skin expressing the molecules they target. For example, EGFRIs have a high frequency of papulopustular eruptions, dry skin, and itching. Furthermore, these symptoms correlate with the poor quality of life experienced by these patients; therefore, a dosage adjustment may be warranted (9–11). Moreover, although immune checkpoint blockade is efficacious, it can produce irAEs, including a range of skin symptoms, from minor rashes to severe and even life-threatening ones (12–14). Causes of these skin toxicities are many and varied and include direct drug toxicity, disruption of the immune system, genetic factors, and environmental factors. In more recent studies, factors such as zinc deficiency exacerbating EGFR-TKI-induced skin reactions and genetic polymorphisms increasing the risk of radiotherapy-induced skin toxicity were found, making personalization of care necessary (9, 15).

The fallouts of skin toxicities are not just physical annoyance, but it affects mental health, social life, and sticking to the treatment plan. Based on the surveys and clinical studies, patients who suffered from severe cutaneous reactions reported negative body image and decline of quality of life. This leads to interruption or discontinuation of the treatment, jeopardizing cancer control (16, 17). Healthcare professionals recognize the clinical importance of such toxicities and are cautious and prompt. However, management guidelines are not clear, and patients not adhering to the preventive measures of skin toxicity need more defined care pathways and better patient education (18).

As gynecological cancers get more complicated to treat, we need fresh, evidence-based methods for handling skin problems that occur while treating the cancer. From 2021 to 2025, there is a rush of research on this topic; it is very important to explore the clinical characteristics, molecular mechanisms, and treatment strategies for treatment-induced skin toxicity. Furthermore, there are progressive radiation methods, such as Hypofractionated Proton therapy, which have the opportunity to decrease skin damage while keeping the quality of the treatment (19). Moreover, AI for forecasting negative reactions and enhancing the preparation of treatments is becoming popular (20). New treatment methods involving nanotech and new medicine vehicles show promise in reducing whole-body harm and alleviating skin discomfort (21). The aim of implementing these advances into the clinic is to improve patient outcomes, and this comes to light by way of finding that middle ground when treating the cancer as well as protecting the skin and the person’s general quality of life.

In summary, gynecologic cancer is complex because of its frequency, the individuality of each patient’s journey, and the burden of treatment side effects. Skin ailments are a common adverse affect related to cancer treatment; more focus is needed on this issue to avoid disruption of therapy and maintain the quality of life of those receiving treatment. Research from 2021 to 2025 has also shown an uptick and provides evidence for a variety of different aspects of these toxicities that guide modern management. This review carefully gathers the most recent knowledge about treatment-induced skin side effects that women’s cancer patients can have to help doctors improve the care they give and the medicines they choose.

## 2 Clinical manifestations and assessment of skin toxicities related to gynecologic cancer treatment

### 2.1 Chemotherapy-induced skin reactions

Chemotherapy, such as paclitaxel, which is used a lot to treat gynecologic cancers, can cause skin toxicities. Paclitaxel belongs to the taxane class, and its subclinical injuries to skin hydration, elasticity, and barrier function have been reported, which can cause clinical manifestations, such as dryness, redness, and peeling (Type I Hypersensitivity). These seemingly minor changes to the skin show that skin balance is changing. Non-invasive exams can give indicators such as raised TEWL (transepidermal water loss), diminished sebum production, and intensified localized inflammation. They can be thought of as quite early signals of a potential issue with the skin’s protective layer (skin barrier) that you may not be able to see or detect yet (overt clinical signs or symptoms). Prompt identification of these markers is important for early intervention to prevent the worsening of skin toxicity. Chemotherapy causes skin damage at the molecular level, reducing important structural and functional proteins, such as AQP3, which keeps skin hydrated, and collagen, which keeps skin whole and stretchy. These molecular indicators show that the chemotherapy is affecting how the outside layers of skin operate and how well they can transport water, meaning it is more difficult for the skin to perform its protective duties. Although there are only a few direct clinical studies of skin biophysical and molecular changes in women with gynecologic cancer who are treated with paclitaxel, more general oncology studies show the same or similar findings to support these interpretations. To understand the subclinical changes, designing preventive and curative methods for chemotherapy-related skin problems would be of use, so that the quality of life of patients is improved and treatment adherence is increased. This is part of a larger trend in oncology to include skin care in overall cancer care because the skin is considered to be an important organ that can be affected by systemic therapy (22).

Chemotherapy-induced dermatologic toxicities frequently include skin rashes, dryness, pruritus, and hand-foot syndrome. Clinical studies examining various chemotherapeutic agents, including 5-fluorouracil and cytarabine, have documented these adverse effects. Typically, these reactions present as maculopapular eruptions or erythema. Their severity is assessed using standardized tools, such as the Common Terminology Criteria for Adverse Events (CTCAE). Management approaches primarily involve the application of topical emollients and adjustments to dosage—including reductions or temporary discontinuation—to mitigate symptoms. However, existing research lacks a comprehensive exploration of the immune microenvironment, specifically the activation, infiltration, and cytokine release of immune cells, which contribute to these dermatologic reactions. This gap exists because the focus has predominantly been on clinical manifestations, their prevalence, and symptomatic management rather than on the underlying immunological mechanisms. Consequently, future studies are urgently needed to investigate the involvement of immune cells, particularly T lymphocytes and macrophages, in the pathophysiology of chemotherapy-induced dermatologic adverse events (23).

### 2.2 Radiotherapy-related skin toxicity

Radiotherapy with high-dose-rate (HDR) brachytherapy is used for gynecological cancers. However, it can cause a variety of skin problems that can affect quality of life and treatment adherence. Inflammation from radiation can cause red, peeling, and puffy skin and usually arises during or close to the radiation period. Alternately, darkish spots might appear, either temporarily or permanently. Chronic Fibrosis is a further significant long-term effect of radiation. Radiation induces the activation of fibroblasts and matrix remodeling. It eventually results in the skin becoming thicker, less elastic, and losing its function. Such adverse effects are more intense for HDR brachytherapy due to high-dose irradiation close to the skin, resulting in severe local tissue damage. These toxicities are closely linked to certain aspects such as total radiation dosage, frequency of dosing, and the specific part of the body being targeted; regions of thin skin or areas prone to mechanical pressure are particularly liable (24, 25).

More recently, new ways of delivering radiotherapy, such as 3D-printing technology to create individualized applicators, have been used to conform the dose to the tumor and reduce exposure to the surrounding healthy tissue. These personalized 3D-printed applicators offer a superior fit to the anatomy and permit precise aiming, and so theoretically, reduce the frequency and severity of skin toxicity by sparing the surrounding healthy skin. However, even with these technologies, there is some clinical evidence that some patients continue to experience severe (grade 3 or greater) skin reactions. Moreover, because this method cannot reduce the amount of radiation needed, we need to consider whether the 3D technology actually improves patient experience, and whether they are actually more efficient at targeting the cancer while alleviating potential side effects. Cancer cases are all individual, making it hard to accurately calculate the side effects any one person will experience (24, 26).

Additionally, patient-specific factors significantly influence the onset and progression of radiation-induced skin toxicity. Different skin types, genetics influencing DNA repair operations, and conditions such as diabetes or blood vessel issues can change how skin responds to radiation exposure. Moreover, concurrent chemoradiotherapy regimens, which are frequently employed in the treatment of gynecological malignancies, are thought to raise skin toxicity due to their synergistic effect on cell harm and lower healing capacity. Tissue volume irradiation and how doses are distributed—pelvic and vulvar areas have lots of skin folds and moisture, which makes dermatitis worse—raises toxicity risks. Verification techniques, such as Cone Beam Computed Tomography (CBCT), are needed to make sure that the dose is delivered correctly and to decrease the chance of accidentally overdosing the skin. However, even with the most precise of imaging techniques, skin reactions have occurred due to a dose difference (25, 26).

The implications of radiation-caused skin issues affect more than patient wellbeing. They could result in radiotherapy being interrupted or stopped altogether, thereby putting the cancer cure at risk. Data from large databases show that treatment breaks because of toxicity are a key reason why radiotherapy is not completed properly, so we need to be on top of how we look after the skin and work with different specialists simultaneously. More complicated chronic wounds or necrosis in or around the vulva or vagina will involve a team of specialists, including radiation oncologists, gynecologic oncologists, and reconstructive surgeons, who will have to cooperate to ensure wound-healing and symptom relief. Different methods, such as improved wound treatment, hyperbaric oxygen treatment, and accurate debridement, have been applied to deal with these hard-to-solve problems with different effects (27, 28).

In conclusion, while advancements in technology, such as HDR brachytherapy and 3D-printed tailored applicators, have been achieved, skin toxicity from radiotherapy in gynecologic cancer patients remains a clinical concern. Skin reactions’ frequency and intensity have complex ties to various elements like the amount of radiation, the total volume of treatment, and personal patient traits such as genetic vulnerability and combined therapy. Continuous improvement of radiotherapy techniques is essential to reduce toxicities, and multidisciplinary approaches are key to improving patient wellbeing.

### 2.3 Targeted therapy and immunotherapy-related cutaneous reactions

Targeted therapies and immunotherapies have changed how we treat cancer, especially when it comes to women’s cancers. However, the application of such therapies is usually accompanied by dermatological adverse effects, which can deteriorate the quality of life of the patients and their compliance with the therapy. Among all these targeted agents, PARP inhibitors such as niraparib are generally thought to have an acceptable safety profile with respect to skin toxicity. Yet, there are isolated occurrences of cutaneous adverse effects, so we need careful ways to manage them that use things to shield the skin from light. Photoprotection must be carried out, since some types of skin toxicity may be aggravated with UV light exposure, and photosensitivity reactions or rash aggravation can be induced. Although data related to the complete skin toxicity caused by PARP inhibitors are still rare, the case report indicates that it is necessary to recognize this kind of rare but severe adverse event. Therefore, some preventive measures, such as patient education and regular dermatological observation, are important to avoid this kind of skin toxicity (29).

Conversely, ErbB family inhibitors—particularly afatinib—frequently trigger dermatological adverse effects. Afatinib is a non-selective, irreversible tyrosine kinase inhibitor of epidermal growth factor receptors (EGFR), HER2, and HER4. Afatinib typically causes cutaneous toxicities such as papulopustular eruption, mucositis, and rash, all of which are dose-limiting. These cutaneous reactions vary in intensity and treatment interruptions are often required because of them, which can negatively affect oncological results. Clinical studies have shown that most patients taking afatinib suffer from skin toxicity, the most prominent ones being acneiform rash and mucosal inflammation. Seriousness of the response is connected to poor quality of life, and could result in worry and withdrawal from society. Management regimens work; they propose early intervention that uses topical corticosteroids, antibiotics, and supportive skincare to stop the symptoms from getting worse. Phase II clinical trials investigating afatinib in patients with HER2-activating mutations reported skin adverse events such as acneiform rash in approximately 30% of the patients and discontinuation of treatment due to adverse events in 11% (29, 30). Furthermore, it is necessary to perform patient education and interdisciplinary treatment, such as dermatology, to effectively manage the condition and be compliant with the treatment. While PARP inhibitors, such as niraparib, have a relatively less intense profile of skin toxicity that can be handled by photoprotection, ErbB family inhibitors often trigger more serious dermatological side effects that require proactive handling to keep the therapy going and maintain a good quality of life for the patients.

The administration of immune checkpoint inhibitors (e.g., PD-1/PD-L1 blockers) frequently induces cutaneous adverse effects, posing significant challenges in clinical management. In gynecological oncology patients receiving these therapies, cutaneous immune-related adverse events (irAEs) manifest in 30–40% of cases, with the predominant presentations being maculopapular eruptions (60.9%), followed by vitiligo (17.4%) and lichen planus-like reactions (10.9%) (31). Moreover, the most life-threatening dermatologic complications, including drug reaction with eosinophilia and systemic symptoms (DRESS) syndrome and Stevens-Johnson syndrome/toxic epidermal necrolysis (SJS/TEN) spectrum disorders, are characterized by mortality mechanisms involving excessive T-cell activation, cytokine release syndrome, and genetic predisposition associated with particular HLA haplotypes (32). Population-based registry analyses demonstrate that immunotherapy-associated severe cutaneous adverse reactions carry significantly elevated fatality rates compared to conventional chemotherapeutic agents, with TEN-associated mortality rates reaching as high as 55.3% (33). Timely recognition and therapeutic intervention are paramount for optimizing clinical outcomes. Current guidelines advocate a multidisciplinary approach incorporating corticosteroid therapy, intravenous immunoglobulin administration, and other supportive measures while balancing oncological efficacy with toxicity management (34) (see Table 1).

**Table 1:** Comparative mechanisms of dermatotoxicity in gynecologic cancer treatments.

Treatment modality	Representative agents /techniques	Clinical phenotype	Molecular & biophysical alterations	Immune microenvironment dynamics (key highlights)
Chemotherapy	Taxanes (e.g., Paclitaxel)	Dryness, desquamation (peeling), erythema, pruritus.	• Downregulation of AQP3 (hydration loss). • Collagen degradation. • Increased TEWL (barrier dysfunction).	• Innate activation: TLR4 signaling in keratinocytes releasing IL-1*β*/TNF-α. • Cellular shift: recruitment of neutrophils/macrophages; Depletion/apoptosis of Langerhans cells. • T-cell Imbalance: reduced Tregs leading to pro-inflammatory dominance.
Radiotherapy	HDR Brachytherapy, EBRT	Acute: erythema, edema, moist desquamation. Chronic: fibrosis, hyperpigmentation, necrosis.	• DNA double-strand breaks. • ROS generation & oxidative stress. • Vascular damage & ischemia.	• Acute phase: massive release of DAMPs; Infiltration of neutrophils & M1 macrophages. • Chronic phase: sustained TGF-β signaling driving fibroblast-to-myofibroblast transition (Fibrosis); M2 macrophage polarization.
Targeted therapy	EGFR inhibitors (Afatinib)	Papulopustular (acneiform) rash, mucositis, paronychia.	• Inhibition of EGFR signaling is essential for keratinocyte survival/differentiation. • Impaired barrier repair.	• Immune-epithelial crosstalk: disrupted antimicrobial peptide production. • Infiltration: predominance of Th17 cells and neutrophils driven by IL-17/IL-23 axis.
PARP inhibitors	PARP inhibitors (Niraparib)	Photosensitivity, rare rash.	• Altered DNA repair signaling. • UV-light interaction.	• Interferon Signaling: Modulation of IFN-related pathways in keratinocytes. • Trigger: UV exposure acts as a stressor activating localized immune responses.
Immunotherapy	PD-1/PD-L1 blockers	•Maculopapular eruptions, vitiligo, lichen planus-like reactions •DRESS, SJS/TEN spectrum disorders	• Genetic predisposition associated with particular HLA haplotypes	• Excessive T-cell activation leading to cytokine release syndrome • Abnormal activation of local immune responses driving cutaneous inflammation and tissue damage

AQP3, Aquaporin-3; TEWL, Transepidermal Water Loss; TLR4, Toll-like Receptor 4; ROS, Reactive Oxygen Species; DAMPs, Damage-Associated Molecular Patterns; TGF-β, Transforming Growth Factor-beta; Tregs, Regulatory T cells; DRESS, Drug Reaction with Eosinophilia and Systemic Symptoms; SJS/TEN, Stevens-Johnson syndrome/toxic epidermal necrolysis.

## 3 Molecular mechanisms and pathophysiological basis of skin toxicity

### 3.1 Molecular effects of chemotherapeutic agents on skin cells

Chemotherapeutic agents targeting rapidly proliferating cancer cells also disrupt healthy skin cells, resulting in treatment-induced dermatologic toxicity. One such agent is paclitaxel, a common chemotherapeutic that can affect how genes are expressed in skin cells and affect the production of collagen and elastin. These proteins are very important to keep your skin healthy and protect it. Collagen and elastin synthesis decreases and the extracellular matrix (ECM) becomes less strong and resilient, which can impact the skin’s barrier function by making it less strong and resilient. In the clinical field, such molecular disruption would be seen as fragile skin, dryness, and a risk of injuries and infections occurring during chemotherapy. Mechanically, paclitaxel causes the dermal cells to be toxic by interrupting their metabolism, cell multiplication, and angiogenesis, affecting DNA replication, transcription, and translation. These kinds of disruptions also affect different cell signaling pathways that are important for making sure that our skin is stable and healthy and able to repair itself (35).

Additionally, deficiencies in DNA repair mechanisms have a significant effect on skin sensitivity to the negative consequences of chemotherapeutic agents. Inherited abnormalities in nucleotide excision repair pathways, for instance, xeroderma pigmentosum (XP), can lead to an enhanced vulnerability toward chemotherapy-caused skin damage. XP has mutations in genes that repair UV-induced DNA damage, causing a build-up of unrepaired DNA damage. When these patients are exposed to chemotherapeutic agents that induce DNA damage (such as alkylating agents and antimetabolites), their skin cells cannot properly fix the damage, which makes the cell toxicity and skin toxicity worse (36). This decreased ability to fix damage makes the adverse effects of chemotherapy on skin cells even worse, potentially leading to more serious problems such as erythema, desquamation, and ulceration. The complex nature of chemotherapy, causing DNA injury and an inherited lack of repair, means that molecular pathways are essential in predicting the level of damage.

Chemotherapeutic agents can activate pro-inflammatory pathways at the molecular signaling level, such as NF-κB and STAT3, which are involved in the inflammatory response seen in the skin. For example, natural substances such as proanthocyanidins have been shown to modulate JAK2/STAT3 signaling and to alleviate chemotherapy-induced thrombocytopenia. This suggests that chemotherapy can impact cytokine signaling cascades, which can then impact skin inflammation and repair (37). In addition, the oxidative stress caused by the high levels of ROS that result from chemotherapy harms the components of skin cells, making them less functional and viable (38). This oxidative damage may cause keratinocyte and fibroblast apoptosis, destroying the structure and immunity of the skin.

Chemotherapeutics such as paclitaxel influence gene expression for collagen and elasticin production in our skin, and they affect DNA repair, which is particularly harmful for at-risk individuals. Individuals with Xeroderma Pigmentosum have cells that get even more damaged because they are genetically pre-disposed to be so. Moreover, they activate the pathways that are related to inflammation and oxidative stress. Together, all of these molecular changes can affect how well a barrier works and lead to skin problems due to chemo. It is crucial to have a complete understanding of these underlying mechanisms to develop specific strategies to prevent or reduce dermatologic adverse reactions during cancer therapy.

### 3.2 Mechanisms of radiation-induced inflammation and fibrosis

While radiation therapy (RT) is a cornerstone of cancer treatment, it frequently induces collateral damage in adjacent healthy tissues. This can result in swelling and subsequent fibrosis, particularly affecting the skin and subcutaneous layers. Much of the damage originates from the radiation, which can cause DNA injury and apoptosis and trigger acute inflammation. An inflammatory response could include immune cells infiltrating and secreting pro-inflammatory cytokines such as tumor necrosis factor-alpha (TNF-α), interleukins (IL-1*β* and IL-6), and transforming growth factor beta (TGF-β). They play critical parts in the orchestration of tissue remodeling and fibrogenesis (39, 40).

Radiation-induced inflammation activates fibroblasts and myofibroblasts, leading to excessive collagen deposition and ECM remodeling, which result in skin fibrosis and functional limitations (41, 42). The core fibrotic process is the transmission of TGF-*β*/Smad, with more radiation resulting in more TGF-β1. Growth factor binds to its respective receptor, which then activates Smad2/3 protein that moves to the nucleus to begin transcription of fibrotic genes, such as collagen and *α*-smooth muscle actin (α-SMA) (43, 44). It does not just promote the proliferation of fibroblasts and their differentiation into myofibroblasts; it also triggers EMT, and this EMT promotes fibrogenesis (45).

In addition, the oxidative stress caused by radiation promotes inflammation and fibrosis through the generation of ROS, leading to cell damage and pro-fibrotic signaling (46, 47). Other molecular players, such as NF-kB and NADPH oxidases, contribute to the ongoing inflammatory milieu (39, 44). More recently, specific pro-resolving mediators and their receptors have been found to play a role in counteracting inflammation and preventing scarring. These results point to some possible treatment directions (40).

These biological pathways interact to drive the progression of cutaneous fibrosis. Histologically, there is more deposition of collagen types I and III, and activated fibroblasts and vascular structures are damaged. It is clinically demonstrated as a hardening, reduced elasticity, and decreased function of the skin (41, 42). This understanding has led to a search for different interventions, including metformin, adipose-derived stem cells, and newer agents targeting the TGF-*β*/Smad and NF-κB pathways to reduce radiation-induced skin toxicity (41, 44). To sum it all up, the skin toxicity that happens after radiation is due to many interacting factors, which can cause reddening and skin sensitivity as well as hardening. TGF-*β*/Smad signaling pathway is essential for the activation of fibroblasts, collagen deposition, and skin fibrosis and dysfunction.

### 3.3 Targeted drug-related immune-mediated cutaneous reactions

Targeted therapies, such as EGFR inhibitors and ICIs, cause immune-triggered skin issues that are hard to deal with in a hospital. EGFR inhibitors mostly cause skin inflammation via immune cell-driven inflammation and keratinocyte dysfunction. The EGFR signaling pathway plays a crucial role in keratinocyte proliferation, differentiation, and survival; blocking it will disrupt the homeostasis of epidermis and affect the barrier function and inflammatory skin symptoms. Immune effector cells, such as T lymphocytes and dendritic cells, get turned on because of the changed signaling from keratinocytes. This activates and triggers the release of a cascade of inflammatory cytokines and chemokines. Clinically, papulopustular eruptions, xerosis, and itch, for instance, rare occurrences of panniculitis and granulomatous skin responses, have been noted in melanoma patients receiving BRAF and MEK inhibitor therapy. These immune-mediated adverse effects highlight the complicated interplay among targeted therapies and host immune reactions (48, 49). The relationship of these immune-mediated reactions with oncologic outcomes is convoluted; there is an interesting paradoxical correlation between immune-mediated reactions and better oncologic outcomes; thus, immune activation is contributing to both adverse effects and benefits.

Immune checkpoint inhibitors, such as antibodies targeting PD-1, PD-L1, and CTLA-4, enhance T cell -mediated antitumor responses. They also impair immune tolerance, which causes a wide range of cutaneous irAEs. The cutaneous toxicities related to ICIs have a range of presentations and can result in inflammatory dermatitis, lichenoid reactions, vitiligo-like depigmentation, bullous pemphigoid, and severe hypersensitivity reactions (50, 51). These reactions occur due to the immune system mistakenly attacking certain parts of the skin, such as melanocytes, which cause the skin to lose its color in something that looks like vitiligo, or proteins in the bottom layer of the skin that cause blisters called bullous pemphigoid. T-cell activation is broadly increased by ICIs in dermatitis-related transcriptomic studies, with several T-cell subsets being affected, as well as both Th1 and Th2 pathways being activated, indicating complex immune dysregulation (52). Moreover, there is the rare but serious risk of immune checkpoint blockade to cause hypohidrosis and autoimmune disease. This shows how important it is to closely monitor and tailor treatment plans (48).

The pathophysiological mechanisms of these immune-mediated skin toxicities involve a failure of peripheral tolerance and the activation of effector T cells, often accompanied by cytokine dysregulation and inflammatory cell infiltration. For instance, Th2 cytokines, such as IL-4 and IL-13, have been linked to bullous pemphigoid related to ICIs. New approaches that target these pathways are looking promising for managing irAEs without harming antitumor immunity (51). In addition to this, paradoxical cutaneous reactions associated with biologics against TNF-α or IL-17 pathway indicate the potential of immunomodulation leading to unanticipated inflammatory skin diseases, such as psoriasiform eruptions and alopecia. This also highlights the delicate balance of immune homeostasis in the skin (53, 54).

In conclusion, targeted therapies cause immune-mediated cutaneous reactions through different yet similar pathways that involve keratinocyte dysfunction, immune cell activation, and cytokine dysregulation. It is necessary to understand the processes to improve the management strategies that reduce the toxicity of the skin. With the anticancer effect kept, some non-invasive diagnosing methods, such as line-field confocal optical coherence tomography (LC-OCT), can help us to detect harmful skin reactions earlier. This could reduce the need for biopsies, which can be dangerous in weak cancer patients (55). Future studies have to figure out the molecular mechanisms causing these negative results and try to find ways to prevent or deal with these immune-caused skin problems without blocking life-saving cancer treatments.

## 4 Risk factors and predictive models of skin toxicity

### 4.1 Patient-related factors

Patient-related variables substantially influence both the chance and strength of treatment-caused skin troubles during the handling of gynecological cancers, especially concerning radiation therapy and targeted therapies. Age turns out to be an important factor, where older people tend to show more sensitivity to skin toxicity. This is related to a diminished ability of the skin to regenerate and changes in the immune system. Moreover, this susceptibility is also made worse due to comorbidities, which are more common in older people and can make it harder for the body to repair damage. For example, an evaluation of more than 749,000 patients by the National Cancer Database has shown that advanced age is related to an independent early termination of radiation treatment. This may be due to the fact that older patients are at a higher risk of developing toxic side effects such as skin reactions (26). This research’s findings also revealed that having a wide range of coexisting disorders and mixing up chemoradiation treatments greatly raises the likelihood of stopping therapy because of adverse effects, highlighting how general health is intricately linked to the degree of skin damage.

Besides patient-related variables, genetic elements that are related to DNA repair deficiencies, such as the mutation of the XP-C gene, greatly affect a person’s susceptibility to radiation-induced skin damage. Mutations in XP-C disrupt nucleotide excision repair. It disrupts the process, leading to a buildup of DNA damage when exposed to UV or ionizing radiation, which increases the danger and seriousness of cutaneous radiation toxicity. There are not many reports from doctors about XP-C gene changes in women with cancer of the womb or other female organs. However, doctors should monitor these patients closely.

Furthermore, pre-existing dermatologic conditions significantly influence the risk of skin toxicity. People with pre-existing skin problems, such as eczema or psoriasis, could find that their skin problems become worse or that the medicine for cancer causes their skin to react in unpredictable ways, making treatment difficult. Furthermore systemic factors, such as anemia and ECOG performance status, are linked to the results of skin toxicity. Suboptimal performance status means there is less energy stored in the body, and it is related to the worsening of skin reactions and less tolerance for cancer treatments. For example, in the clinical trial of afatinib, an ErbB family tyrosine kinase inhibitor, patients with a performance status of ≤1 were selected so as to reduce the impact of the confounding variable of toxicity. However, toxicity-related side effects, such as acneiform rash and mucositis, were quite typical, and 11% of patients had to stop the treatment because of toxicity (29). This finding highlights the pressing need to evaluate baseline functional status as a predictor of skin toxicity.

Socioeconomic factors, though not patient-specific, can also affect how likely an individual is to develop certain illnesses and interact with patient-related variables, indirectly affecting the risk of skin toxicity via their influence on treatment adherence and access to support. Lower income individuals and those with non-private insurance had a higher rate of radiation therapy discontinuation due to toxicity. This suggests that systemic factors can increase the clinical consequences of skin toxicities (26).

In conclusion, patient-related factors such as advanced age, impaired DNA repair (e.g., XP-C mutations), presence of pre-existing dermatologic conditions, anemia, and poor PS significantly impact risk and severity of skin toxicities during the treatment of gynecologic cancer. These variables need custom risk appraisals and supportive care plans to improve the tolerability of treatment and overall patient outcomes.

### 4.2 Treatment-related factors

Doses of chemotherapy and radiotherapy, as well as their combination, are critical elements that affect the incidence and severity of chemotherapy-related skin toxicities in women with gynecological cancers. Agents such as paclitaxel, which is commonly used in gynecologic oncology, are known to produce subclinical changes in skin physiology without any obvious clinical signs. Paclitaxel reduces skin hydration, increases transepidermal water loss, reduces sebum secretion, decreases skin elasticity and skin thickness, and causes erythema, skin roughness, and desquamation. These changes show that there are molecular changes in skin markers, such as aquaporin 3, collagen type 1, elastin, and fibronectin. All of these biomarkers are required to protect the skin from damage and to keep a barrier in place. This means that the clinical settings might not totally realize the chemo-related skin toxicity because it is beneath the radar, which makes careful watch over dose administration and skin parameters important throughout the treatment procedure (56).

Furthermore, radiotherapy dosage and fractionation schemes can significantly influence the profile of skin toxicity. For example, in definitive radiotherapy–chemotherapy protocols for vaginal carcinoma, median external beam radiotherapy doses of approximately 45 Gy and brachytherapy doses approaching 28 Gy are used, which have been applied with tolerable acute and chronic skin toxicities. However, there are some extremely rare cases of acute grade 3 skin toxicity, indicating that a high cumulative dose of radiation or more intense treatments might increase the risk of severe skin side effects (57). In addition, retrospective analyses of chemoradiotherapy for cervical cancer have found that even as contemporary radiotherapy improves survival rates, acute and late grade 3–4 toxicities, such as skin reactions, still affect many patients. These observations underscore the need to optimize the dose of chemotherapy and radiotherapy to obtain the appropriate balance between efficacy and toxicity (58).

Additionally, the combination of chemo and radiation (chemoradiotherapy) is independently associated with a higher risk of discontinuation of radiotherapy, which may be partially attributed to toxicity, including skin-related toxicities. Analysis of data from national databases shows that patients undergoing chemoradiation are more likely to stop their chemoradiation early, indicating the clinical relevance of treatment-related toxicities to the adherence to the therapy and overall patient outcomes (26). In short, the findings show that the strength of the chemotherapy dosage, the level of radiotherapy dosage, and their combination are major factors in the incidence and severity of skin toxicity in treating gynecological cancers, demanding individualized changes in dosage and care methods for supporting therapy to reduce negative skin reactions.

After the treatment-related toxicity, the use of 3D-printed applicators for HDR brachytherapy in the context of gynecological malignancies is an important technological advance for the optimization of dose conformity and protection of normal tissues. Clinical outcomes of recent studies using 3D-printed applicators show encouraging local control rates with tolerable toxicity in both primary and recurrent gynecological cancer cases. Take the group of patients who were given the treatment using the in-house developed 3D-printed applicator as an example, the complete response rate could reach up to 92.9% at the end of 3 months after the treatment, and for the primary cases, the local relapse-free survival rate for 2 years reached 100%. Even though the oncological results were positive, skin and mucosal toxicity remained an issue, with nearly 28.6% of people having acute Grade 3 skin toxicity, and a lesser portion getting ongoing Grade 3 vaginal toxicity (59).

A superior dose distribution from the 3D-printed applicators might be able to better adjust to the different body parts for each individual, and thereby reduce the dosage of radiation on organs at risk. The problem of skin dose is further compounded by immobilization devices used for IMRT, such as the combination of the prone immobilization device and the belly board, where the skin dose was recorded as having been significantly increased due to the use of immobilization devices; an increase of up to 23.79% in skin doses was recorded in the case of using the non-immobilization device calculation. These kinds of discrepancies in dosage show the need for an immobilization device as a part of the treatment planning contours, to have an exact estimation and reduction of the skin dose.

While 3D-printing technology significantly improves dose accuracy and personalization in gynecological brachytherapy, its impact on skin dose must be thoroughly understood. It is quite important to integrate strategies for spotting and handling skin problems that might come up while treating cancer into the plan for both dealing with the cancer and giving the treatment. It comprises exact dose computations, taking into account the effect of applicators and immobilization apparatus on dose distribution, as well as performing proactive skin care actions. An all-encompassing plan aims for the best form of treatment and cutting down on skin damage, which would be beneficial for the patient both during and after their treatment.

### 4.3 Socioeconomic and healthcare service factors

The socioeconomic status of an individual and the kind of healthcare services used are important aspects that impact the management of treatment-related toxicities and continuation of cancer treatment, especially in the field of gynecologic oncology. Comprehensive analysis of data from the National Cancer Database, which contained 749,135 patients who underwent RT or combined treatments, showed a significant impact of insurance, income, and facility characteristics on adherence to treatment and managing toxicities (26). Specifically, patients with non-private insurance, e.g., Medicaid or uninsured, and lower-income groups have a higher probability of stopping RT prematurely. This trend is often due to toxicity reasons or patients deciding to stop taking the drug. The results show that financial and insurance-related issues can make it hard to get constant and full care for treatment, which means people might not finish their treatment.

Being treated at a community-based healthcare facility instead of an academic or integrated cancer center was independently linked to more disruption of treatment. It might point to the fact that some people get access to different resources, such as certain kinds of workers who can see and help fix early signs of something going wrong, or people who know how to teach and help patients in different ways. Adequately resolving skin issues from oncologic treatment is influenced by the healthcare resources available and the level of education the patient has. Those who are more socially and economically privileged and who have easy access to quality healthcare institutions will most likely receive timely interventions for their skin toxicity, leading to higher chances of adhering to treatment and enjoying an overall enhanced quality of life due to lessened severity in their skin toxicity. Alternately, lower-income patients might not be aware of whether a side effect is abnormal or how to address it, so they have a much higher chance of having negative side effects and giving up on the treatment. Therefore, assuming economic and social aspects and improving healthcare services–especially for those who cannot reach them–is necessary to make the treatment of skin toxicities better in this group of cancer patients. Improving insurance provision, educating patients on potential side effects, and ensuring fair access to treatment would all improve cancer care (26).

## 5 Comprehensive management and innovative strategies for skin toxicity in gynecological tumors

### 5.1 Basic prevention and skin barrier care

Preventive skin care is fundamental to the treatment of gynecological malignancies, as it preserves the skin barrier and blocks the inflammatory cascade triggered by chemotherapy and radiotherapy. For patients receiving pelvic radiotherapy or systemic chemotherapy, early damage to the skin barrier often occurs before visible dermatitis, showing up as drier skin and increased transepidermal water loss (TEWL). Clinical studies strongly support the introduction of emollient interventions early in treatment: for example, emollients containing ingredients such as olive oil, urea, or hyaluronic acid can significantly delay the onset of radiation dermatitis and reduce its severity by replenishing stratum corneum intercellular lipids and enhancing stratum corneum hydration (60). More advanced physical protective measures, such as the preventive use of polyurethane films (e.g., Hydrofilm), have been shown to be very effective in clinical trials. These semi-permeable dressings not only provide a moist healing environment for damaged skin but also serve as a physical barrier to reduce mechanical irritation from clothing friction, thereby significantly alleviating radiation-induced erythema, pigmentation, itching, and pain. Due to their minimal side effects and good patient compliance, this preventive barrier strategy is becoming one of the standard procedures in the care of radiation dermatitis (61).

In addition to repairing physical barriers, proactive defense against environmental factors—especially sun protection and preventive medications—is equally crucial. Many chemotherapy drugs used to treat gynecological tumors (such as paclitaxel and doxorubicin) and molecular-targeted therapies (such as EGFR inhibitors) exhibit significant photosensitivity, increasing the skin’s sensitivity to UV radiation and leading to severe erythema or pigmentation. Although some chemical sunscreens may raise concerns about environmental or endocrine disruption, for cancer patients, the use of approved broad-spectrum sunscreen to prevent additional UV damage is a clinically necessary measure with far more benefits than drawbacks (62). Furthermore, with the widespread use of targeted therapies, skin-related preventive strategies have extended to the pharmacological level. For instance, during the use of EGFR inhibitors, simple skincare often proves insufficient to curb the outbreak of rashes, whereas combining oral preventive antibiotics (such as doxycycline or minocycline) with standardized skincare regimens has been shown in multiple clinical trials to effectively suppress bacterial colonization of hair follicles and neutrophil chemotaxis—the process by which neutrophils are attracted to sites of inflammation—thereby reducing the incidence of moderate to severe rashes by over 50%. Coupled with real-time monitoring of skin biophysical parameters (such as TEWL values) using appropriate devices, clinicians can identify signs of barrier dysfunction earlier, allowing for intensified interventions before visible rashes occur (63).

### 5.2 Advances in drug treatment and novel delivery systems

When basic preventive measures fail to halt the progression of skin toxicity, pharmacological interventions become necessary to manage symptoms and avoid treatment interruptions. Current standard clinical treatments primarily rely on topical corticosteroids and non-steroidal anti-inflammatory drugs (NSAIDs), which quickly alleviate acute inflammation and swelling by inhibiting phospholipase A2 and cyclooxygenase pathways. However, long-term use of potent corticosteroids may lead to side effects such as skin atrophy and telangiectasia, prompting clinicians to turn to safer alternatives, such as calcineurin inhibitors (tacrolimus and pimecrolimus). These medications specifically block T-cell activation, providing anti-inflammatory effects without affecting collagen synthesis. This makes them particularly suitable for long-term maintenance therapy in delicate skin areas such as the vulva.

Beyond traditional medications, natural compounds with multiple pharmacological activities, including Diallyl disulfide biological preparations, are emerging. For example, Diallyl disulfide extracted from garlic not only exhibits powerful antioxidant and anti-inflammatory properties, protecting keratinocytes from chemotherapy-induced oxidative stress, but has also been found to have potential synergistic anti-tumor effects (64). This dual benefit mechanism of both preventing skin toxicity and exerting anti-cancer effects provides an attractive direction for developing new skin protective agents.

Even more exciting advancements are seen in drug delivery technology, particularly in smart delivery systems based on biomaterials and nanotechnology, which fundamentally address the problems of poor absorption with topical treatments and high toxicity in systemic administration. Hyaluronic acid (HA), due to its natural biocompatibility and targeting of CD44 receptors, is widely used to construct hydrogels and microneedle systems, serving not only as a carrier for sustained drug release but also as a promoter of wound healing and tissue regeneration (65). At the nanoscale, smart nanocarriers synthesized using polymer-induced self-assembly (PISA) technology can respond to unique acidic pH or temperature changes in the tumor microenvironment or inflammatory sites; they undergo structural disintegration to precisely release drugs (66, 67). This stimulus-responsive mechanism ensures that drugs accumulate at high concentrations only at the lesion site, greatly reducing collateral damage to normal tissues. Additionally, films made from chitosan and other natural substances can form breathable drug reservoirs on the skin surface—reservoirs that continuously release anti-inflammatory factors while physically blocking external stimuli (68–70). Meanwhile, microbubble carriers in combination with ultrasound technology represent the cutting edge of non-invasive treatments. These carriers utilize the enhanced permeability and retention (EPR) effect of tumor tissue to achieve targeted accumulation. Subsequently, external ultrasound activates microbubble cavitation, releasing drugs locally. This process is expected to significantly increase drug concentration at the tumor site while minimizing systemic toxicity (71).

### 5.3 Precision physical protection in radiotherapy: 3D printing technology

The introduction of 3D printing technology in the field of radiotherapy represents a big step from the use of generic to custom-made skin protection, effectively solving the dosimetric challenges posed by traditional bolus materials due to poor fit. In traditional radiotherapy practice, standard bolus materials often fail to perfectly conform to complex body areas such as the breast, vulva, or groin, resulting in air gaps that can cause radiation scattering; this leads to insufficient surface doses—increasing the risk of tumor recurrence—or local hotspots—resulting in severe dermatitis. 3D printing technology can utilize patients’ CT data to construct personalized bolus materials that match the body contour to within submillimeter precision. Studies show that 3D-printed bolus can stabilize the air gap between the bolus and the skin to within 1 mm, virtually eliminating the risk of dose non-uniformity (72, 73). Clinical dosimetry comparative studies indicate that using 3D-printed bolus materials in breast cancer and gynecological tumor radiotherapy not only improves the dose coverage of the planning target volume (PTV) but also optimizes radiation pathways, reducing the average dose received by critical organs at risk (OARs) such as the heart and lungs by approximately 0.8 Gy (74). This precise dose modulation directly translates into a decrease in the incidence of acute skin toxicity in clinical settings, allowing patients to complete the full course of treatment more smoothly.

Beyond bolus materials, 3D-printing technology offers promising advancements in manufacturing brachytherapy applicators, particularly for gynecological cancer patients with unique anatomical variations. By printing applicators that perfectly fit the patient’s vaginal morphology, doctors can ensure that the radiation source is always accurately positioned, achieving high-dose irradiation of the tumor target area while allowing the dose to drop rapidly outside the target area—maximizing protection for the rectum, bladder, and surrounding healthy vaginal mucosa (75). Currently, materials research is focused on further optimizing the performance of printed materials. For example, researchers are developing thermoplastic elastomers (TPEs) that combine flexibility and biocompatibility to reduce mechanical damage to mucosa caused by rigid materials (76). More cutting-edge attempts include integrating ultrasound transmission gel channels within 3D-printed structures; this integration eliminates tiny air gaps and enhances the precision of ultrasound-guided delivery (77). Although more data are needed to support material durability and long-term safety, these innovations undoubtedly provide powerful physical solutions for reducing acute and chronic skin toxicity related to radiotherapy (78, 79).

### 5.4 Genomics-guided individualized management and multidisciplinary collaboration

With the advancement of precision medicine, the management of skin toxicity in gynecological tumors is undergoing a paradigm shift from empirical to personalized medicine based on genetics. Utilizing next-generation sequencing (NGS) and multi-omics analysis technologies, clinicians can now analyze patients’ genetic profiles to identify specific molecular subtypes, such as the POLE ultramutated and MSI-H molecular subtypes of endometrial cancer (80, 81). This analysis informs the development of cancer treatment plans and reveals the characteristics of the patient’s skin immune microenvironment. Consequently, it enables the prediction of the risk of severe skin toxicity from immune checkpoint inhibitors or targeted drugs (82). For example, certain gene polymorphisms may exacerbate DNA repair deficiencies, leading to a higher likelihood of severe radiation dermatitis during chemoradiotherapy (83). Combined with AI-driven big data analysis models, medical teams are expected to construct a patient-specific “toxicity risk map” before treatment begins. This approach facilitates identifying high-risk patients and formulating personalized skin protection strategies that include prophylactic dose adjustments, enhanced monitoring, or prophylactic medications, thereby truly achieving “tailored treatment” (84).

This complex management approach requires close interdisciplinary collaboration to be effectively implemented. Dermatologists, medical oncologists, radiation oncologists, clinical pharmacists, and specialized nurses should overcome disciplinary barriers and establish regular molecular tumor boards (MTB) and interdisciplinary outpatient clinics (85, 86). This mechanism is particularly crucial for managing life-threatening severe skin adverse reactions, such as Stevens-Johnson syndrome (SJS) or toxic epidermal necrolysis (TEN), which are classified as Type IVc cytotoxic T-cell-mediated hypersensitivity. Managing these conditions ensures that patients receive comprehensive treatment—from drug adjustments and wound management to systemic support—at the earliest possible time (87–89). At the same time, patient education has been recognized as an essential component of treatment interventions. Studies have shown that proactive consultations by pharmacists and nurses, multimedia video education, and culturally tailored educational materials can significantly enhance patients’ early recognition of symptoms such as skin dryness and erythema (90, 91). This increased patient empowerment enables patients to become active participants in managing adverse reactions, enabling timely reporting of any changes in their condition. This helps avoid unplanned interruptions in anti-tumor treatment due to severe skin toxicity, ultimately improving quality of life and thereby contributing to improved survival outcomes (92–95) (see Table 2).

**Table 2:** Risk factors and management strategies for treatment-related skin toxicities.

Treatment category	Key risk factors (patient & treatment related)	Clinical impact of toxicity	Prevention & management strategies
Chemotherapy (Taxanes)	• Pre-existing dry skin or eczema. • Genetic susceptibility to barrier weakness.	• Subtle subclinical changes may precede overt dermatitis. • Can affect patient comfort and compliance.	• Early detection: Monitoring biophysical markers (e.g., TEWL). • Barrier repair: Intensive moisturizing to support AQP3/collagen function. • Anti-inflammatory: Topical agents to dampen innate immune activation.
Radiotherapy	• Dosimetry: high total dose, bolus effect. • Anatomy: skin folds (vulvar/inguinal), obesity, thin skin areas. • Comorbidities: diabetes, vascular disease, smoking. • Concurrent chemo: “radio-sensitizing” effect.	• Treatment interruptions due to Grade 3 + toxicity. • Chronic wounds/necrosis requiring surgical intervention.	• Technological: use of 3D-printed personalized applicators to spare healthy tissue. • Verification: CBCT image guidance. • Wound care: gentle cleansing, specialized dressings, multidisciplinary care (surgeons/derms).
Targeted & Immunotherapy	• Dose intensity: higher doses of EGFRi correlate with rash severity. • UV exposure: Specifically for PARP inhibitors. • Skin type: variations in photosensitivity.	• Physical disfigurement leading to psychosocial distress. • Potential dose reduction or discontinuation of life-prolonging therapy.	• Proactive: pre-emptive antibiotics (tetracyclines) & topical steroids for EGFRi. • Photoprotection: strict sun avoidance and sunscreen use for PARP inhibitors. • Education: patient counseling on early symptom reporting.

## 6 Conclusion

In summary, managing the skin toxicities associated with gynecological cancer therapies is a complex clinical challenge, as they manifest in various forms and arise from diverse molecular mechanisms. From 2021 to 2025, the research situation has changed from existing research to predicted results. This means that it subclinical skin injury is increasing. These skin injuries are often overlooked and need more medical attention. Early discovery and prompt intervention are good ways to reduce skin problems. This can improve the quality of life for those people being treated for cancer and those who have received treatment for cancer.

Accounting for the heterogeneity of current evidence, a personalized risk stratification framework integrated with a multidisciplinary care model constitutes the fundamental paradigm for skin toxicity mitigation in cancer care. Dermatologists, medical oncologists, radiation oncologists, and interdisciplinary healthcare teams must engage in collaborative practice, as the broad spectrum of patient-specific sensitivities necessitates tailored diagnostic workflows and individualized management algorithms. Accordingly, therapeutic approaches that encapsulate core medical principles while advancing precision oncology align with the evolving paradigm of modern cancer therapeutics.

In addition, when considering newly available therapies, such as 3D-printed radiotherapy applicators and genomes technologies, it is clear we are starting a new epoch where we can prevent and treat these diseases much easier. These tools give us some hopeful chances to avoid skin damage from more precise aiming and dosing, that is, custom body-based fixes. However, we need to have real evidence from clinical trials that these technologies are effective, safe, and cost-efficient.

Looking ahead, further elucidation of the mechanistic pathophysiology governing skin toxicity in gynecologic oncology is warranted. Dissecting the molecular, cellular, and signaling pathways underlying these adverse events will facilitate the development of targeted symptomatic relief strategies. Leveraging this enhanced mechanistic understanding, novel interventional modalities—beyond traditional pharmacological agents—should be explored, including bioengineered skin substitutes and analogous tissue-engineered approaches. These strategies have the potential to improve patients’ holistic well-being and optimize long-term oncological outcomes in gynecologic cancers involving distinct anatomical loci.

Synthesizing our current insights with expert consultations, success in managing skin toxicities within gynecologic oncology will require early detection, personalized treatment plans, multidisciplinary collaboration, and the strategic use of technology. This frame may change how we support patients, improving cancer care and patient wellbeing.

## Data Availability

The original contributions presented in the study are included in the article/supplementary material, further inquiries can be directed to the corresponding authors.
